# The ankle brachial index exhibits better association with cardiovascular outcomes than interarm systolic blood pressure difference in patients with type 2 diabetes

**DOI:** 10.1097/MD.0000000000015556

**Published:** 2019-05-13

**Authors:** Liang-Yu Lin, Chii-Min Hwu, Chia-Huei Chu, Justin G.S. Won, Harn-Shen Chen, Li-Hsin Chang

**Affiliations:** a Division of Endocrinology and Metabolism, Department of Medicine, Taipei Veterans General Hospital; b Faculty of Medicine, National Yang-Ming University; c Division of Otology, Department of Otorhinolaryngology-Head and Neck Surgery, Taipei Veterans General Hospital, Taipei; d Division of Endocrinology and Metabolism, Department of Medicine, Tao-Yuan Branch of Taipei Veterans General Hospital, Tao-Yuan, Taiwan.

**Keywords:** ankle brachial index, cardiovascular outcomes, interarm systolic blood pressure difference, type 2 diabetes mellitus

## Abstract

Increased interarm systolic blood pressure difference (IASBPD) is associated with cardiovascular prognosis in the general population. This study aimed to evaluate whether IASBPD or ankle brachial index (ABI) is strongly associated with cardiovascular outcomes in patients with type 2 diabetes.

Total 446 type 2 diabetes followed up for a mean 5.8 years divided by ABI (<0.9 vs ≥0.9) or IASBPD (<10 vs ≥10 mm Hg). The primary outcome was a composite of all-cause mortality, hospitalization for coronary artery disease, nonfatal stroke, carotid, or peripheral revascularization, amputations, and diabetic foot syndrome. The secondary endpoint was all-cause mortality.

Sixty-four composite events and 17 deaths were identified. The primary and secondary outcomes were higher than those in the group with ABI < 0.9 vs ABI ≥ 0.9 (32.8% vs 11.6%, *P* < .005 for primary outcome; 14.0% vs 2.3%, *P* < .005 for all-cause mortality) but IASBPD cannot exhibit a prognostic value. ABI < 0.9 was also the dominant risk factor of both endpoints demonstrated by multivariate Cox proportional analysis (composite events: adjusted hazard ratio [HR], 2.39; 95% confidence interval [CI], 1.26–4.53; *P* = .007; all-cause mortality: adjusted HR, 3.27: 95% CI, 1.91–5.60; *P* < .001).

The ABI was more associated with cardiovascular outcomes in patients with diabetes than IASBPD.

## 1 Introduction

Diabetes mellitus affected 415 million individuals in 2015 worldwide, and the number is expected to increase to 642 million by 2040 with the increase of economic burden.^[1]^ Type 2 diabetes mellitus (T2DM) is associated with chronic complications related to the impairment of macro- and microvascular beds, which can be well explained by detrimental influences on vascular biology. The relative risk of all-cause mortality in subjects with diabetes were estimated by Emerging Risk Factors Collaboration which including 689,300 participants and showed the all-cause mortality of T2DM were double and equivalent to those with history of ischemic stroke or myocardial infarction.^[2]^ Furthermore, approximately three-fourth of patients with type 2 diabetes die of cardiovascular disease-related events and appears to have little or no excess risk of major adverse cardiovascular events (MACE) compared with the general population.^[3,4]^ In addition, the 18-year-follow-up population-based study in Finland showed that patients with type 2 diabetes had a 1.9-fold increased risk of mortality caused by coronary artery disease (CAD) compared with those without diabetes and without a prior history of CAD.^[5]^

According to the reports of National Health and Nutrition Examination Survey, Patients with type 2 diabetes have a 2.7-fold increased risk of peripheral arterial disease (PAD) compared with the general population.^[6]^ The difference in systolic blood pressures between arms is associated with PAD, cerebrovascular disease, and increased cardiovascular and all-cause mortality.^[7]^ Interarm systolic blood pressure difference (IASBPD) of ≥10 mm Hg has been correlated with cardiovascular events in the general population and in patients with chronic kidney disease or vascular disease.^[7–9]^ The prevalence of IASBPD more than 10 mm Hg between arms in patients with diabetes is 9% to 10% in the previous reports.^[10,11]^ Risk of target organ damage of the kidney, retina, and heart was increasing concordant with higher IASBPD and the IASBPD was also associated with cardiovascular mortality.^[12,13]^

Ankle brachial index (ABI), measured as a ratio of ankle-to-arm systolic blood pressure, is also a validated noninvasive procedure that is used to diagnose PAD. Lee et al showed that an ABI < 0.9 increased the credibility of the model for predicting the risk of fatal myocardial infarction after adjusting for conventional risk factors in the Edinburgh Artery Study.^[14]^ However, the association between ABI and cardiovascular outcomes in general populations was not consistent to the patients with diabetes. A population-based study showed that the association of ABI with all-cause mortality and with cardiovascular mortality was similar regardless of a history of diabetes after a prospective follow-up for 19 years.^[15]^ Furthermore, Mostaza et al demonstrated that ABI was associated with MACE and all-cause mortality patients without diabetes but the association was not found in those with diabetes.^[16]^ The controversial association between ABI and prognosis in patients with diabetes highlights the need for alternative methods of assessing vascular disease and predicting outcomes of diabetes.

The present study aimed to assess the association of ABI and IASBPD with cardiovascular outcomes, and to determine whether ABI or IASBPD is better associated with cardiovascular outcomes in patients with diabetes.

## 2 Materials and methods

### 2.1 Patient population and clinical data

This retrospective chart review study was conducted in accordance with the principles of Declaration of Helsinki and Title 45, US Code of Federation Regulations, Part 46, Protection of Human Subjects, revised November 13, 2001, effective December 13, 2001. Ethical approval was obtained from the Ethics Committee of Taipei Veterans General Hospital, Taiwan. The demographic and anthropometric characteristics, history of CAD or cerebrovascular disease, ABI, and medications in patients with T2DM who were registered in the diabetes share-care program of the division of endocrinology and metabolism of Taipei Veterans General Hospital from July 15, 2005 to December 31, 2007 were reviewed. All measurements of the ABI and IASBPD simultaneously were recorded by an Omron noninvasive vascular screening device (VP-1000; Omron Matsusaka Company, Kubocho, Japan) for screening of PAD. Body mass index was calculated as the weight (kg) divided by the square of height (meters). The IASBPD was defined as the difference of systolic blood pressure between the arms. The laboratory results within 3 months before or after ABI measurement, including hemoglobin A1C level, serum creatinine level, estimated glomerular filtration rate (eGFR) calculated using the Modification of Diet in Renal Disease formula, lipid profiles, and 2 consecutive daily urinary albumin excretions, were recorded.

### 2.2 Groups and research endpoint definition

The enrolled patients were divided into 2 groups based on ABI (≥ or <0.9) or IASBPD (≥ or <10 mm Hg). The clinical outcomes from ABI measurement up to August 2012 were retrospectively reviewed. The primary endpoint was a composite of all-cause mortality, hospitalization for CAD, stroke, carotid or peripheral revascularization, lower limb amputation, and hospitalization for diabetic foot syndrome. The secondary outcome was the all-cause mortality in the different groups.

### 2.3 Statistics

Continuous variables were demonstrated as mean ± standard deviation and the independent analysis was used to comparing the variance. Categorical variables were presented as numbers and portions which were compared by the method of Pearson Chi-squared test. The correlation between ABI and IASBPD was tested by the method of liner correlation and the correlation between ABI < 0.9 and IASBPD ≥ 10 mm Hg was examination by kappa coefficient. The primary and secondary endpoints were analyzed by Kaplan–Meier analysis and the cumulative event-free probabilities of each group were compared by the log-rank test. The univariate Cox proportional analysis was used to identify all relevant variables and the *P* < .1 is the criteria for further subjecting to multivariate Cox proportional analysis which the hazard ratio (HR) as well as corresponding probability values was calculated. The SPSS software package (version 18; IBM Corporation, Armonk, NY) was used for the above analysis.

## 3 Results

### 3.1 Baseline characteristics

We analyzed the medical records of 446 patients with T2DM who were followed up of 3.6 to 6.1 years and the mean duration was 5.8 years (standard deviation 0.5 year). All baseline characteristics of the patients in the 2 groups divided by either ABI or IASBPD are listed in Table 1. The groups with ABI ≥ 0.9 and ABI < 0.9 consisted of 388 and 58 patients, respectively. The groups with IASBPD ≥ 10 mm Hg and IASBPD < 10 mm Hg consisted of 359 and 87 patients, respectively. After grouping based on ABI, the proportion of men and smoking, daily urine albumin excretion, HbA1c level, eGFR, and use of antiplatelets were higher and diabetes duration was longer in patients with ABI < 0.9. After grouping based on IASBPD, body mass index and systolic blood pressure were higher, but ABI was lower in the group with IASBPD ≥ 10 mm Hg. The result of linear correlation between ABI and IASBPD was significant (correlation coefficient = −0.245, *P* < 0.001). Furthermore, the correlation between ABI < 0.9 and IASBPD ≥ 10 mm Hg was also significant (Kappa coefficient 0.093, *P* = 0.043).

**Table 1:**
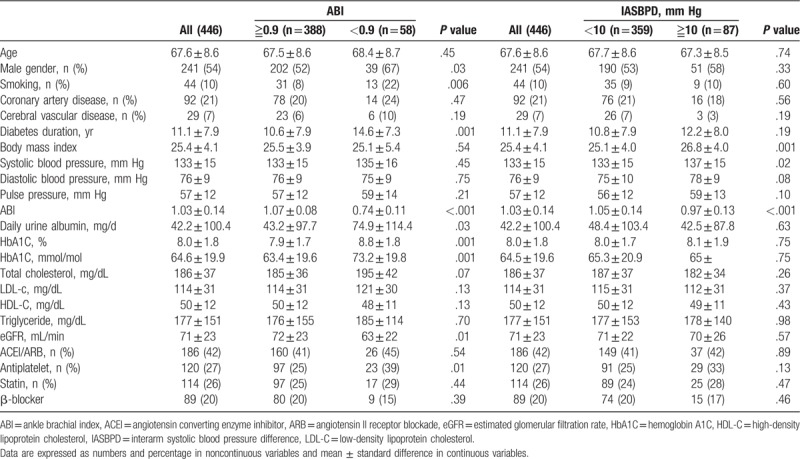
Baseline characteristics of subjects with type 2 diabetes grouped by ABI and IASBPD.

### 3.2 Primary and secondary outcomes

**Figure 1:**
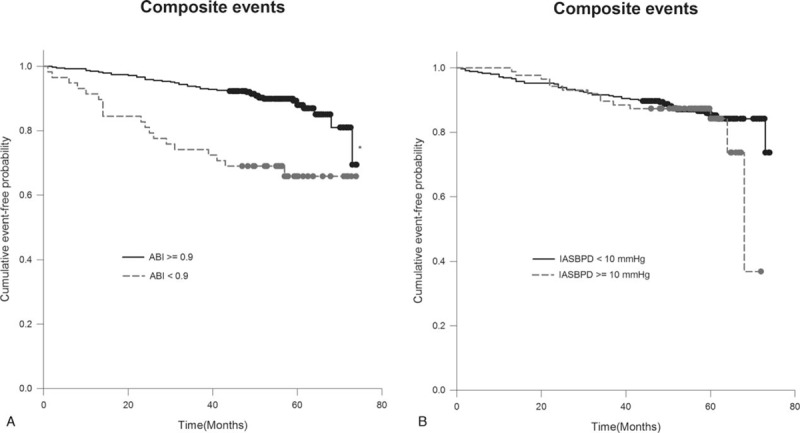
Kaplan–Meier curve showing the cumulative event-free probability of composite events in patients with diabetes grouped by ankle brachial index (ABI) (≥0.9 vs <0.9) (A) and interarm systolic blood pressure difference (IASBPD) (≥10 vs <10 mm Hg) (B). ∗*P* < .001 compared to patients with ABI ≥ 0.9.

**Figure 2:**
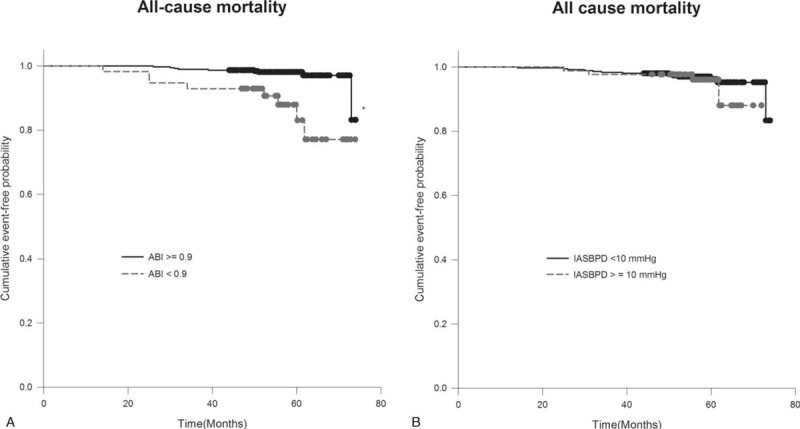
Kaplan–Meier curve shows the cumulative event-free probability of all-cause mortality in patients with diabetes grouped by ankle brachial index (ABI) (≥0.9 vs <0.9) (A) and interarm systolic blood pressure difference (IASBPD) (≥10 vs <10 mm Hg) (B). ∗*P* < .05 compared to patients with ABI ≥ 0.9.

A total of 64 composite events among study subjects were recorded during the follow-up period. The patients with an ABI of <0.9 had higher composite event rates than those with an ABI ≥ 0.9 (log-rank test: 32.8% vs 11.6%, *P* < .005) (Fig. 1A). No significant difference in composite events was observed between patients with IASBPD < 10 mm Hg and those with IASBPD ≥ 10 mm Hg in this analysis (log-rank test: 16.1% vs 13.9%, *P* = 0.560) (Fig. 1B). Similarly, the all-cause mortality rate in patients with ABI < 0.9 was worse than that in patients with ABI ≥ 0.9 (log-rank test: 14.0% vs 2.3%, *P* < .005) but this was not observed when the patients were grouped by IASBPD ≥ 10 or <10 mm Hg (log-rank test: 4.6% vs 3.6%, *P* = .555) (Fig. 2A, B).

### 3.3 Univariate and multivariate regression analyses of ABI and IASBPD

Table 2 lists the results of the univariate Cox proportional analyses between variables and composite events or all-cause mortality. The presence of ABI < 0.9, older age, pulse pressure, diabetes duration, smoking history, history of CAD, eGFR and cholesterol level were significantly associated with composite events. ABI < 0.9, older age, pulse pressure, diabetes duration, history of CAD, and eGFR were significantly associated with all-cause mortality. After adjusting for age, pulse pressure, diabetes duration, smoking history, eGFR, and low-density lipoprotein cholesterol level, ABI < 0.9 was the dominant factor associated with composite events (HR, 2.39; 95% confidence interval [CI], 1.26–4.53, *P* = .007) and all-cause mortality (HR, 6.27; 95% CI, 2.06–19.04, *P* = .001) (Table 2). In contrast, IASBPD ≥ 10 mm Hg showed no association with composite events or all-cause mortality in univariate or multivariate analysis (Table 2).

**Table 2:**
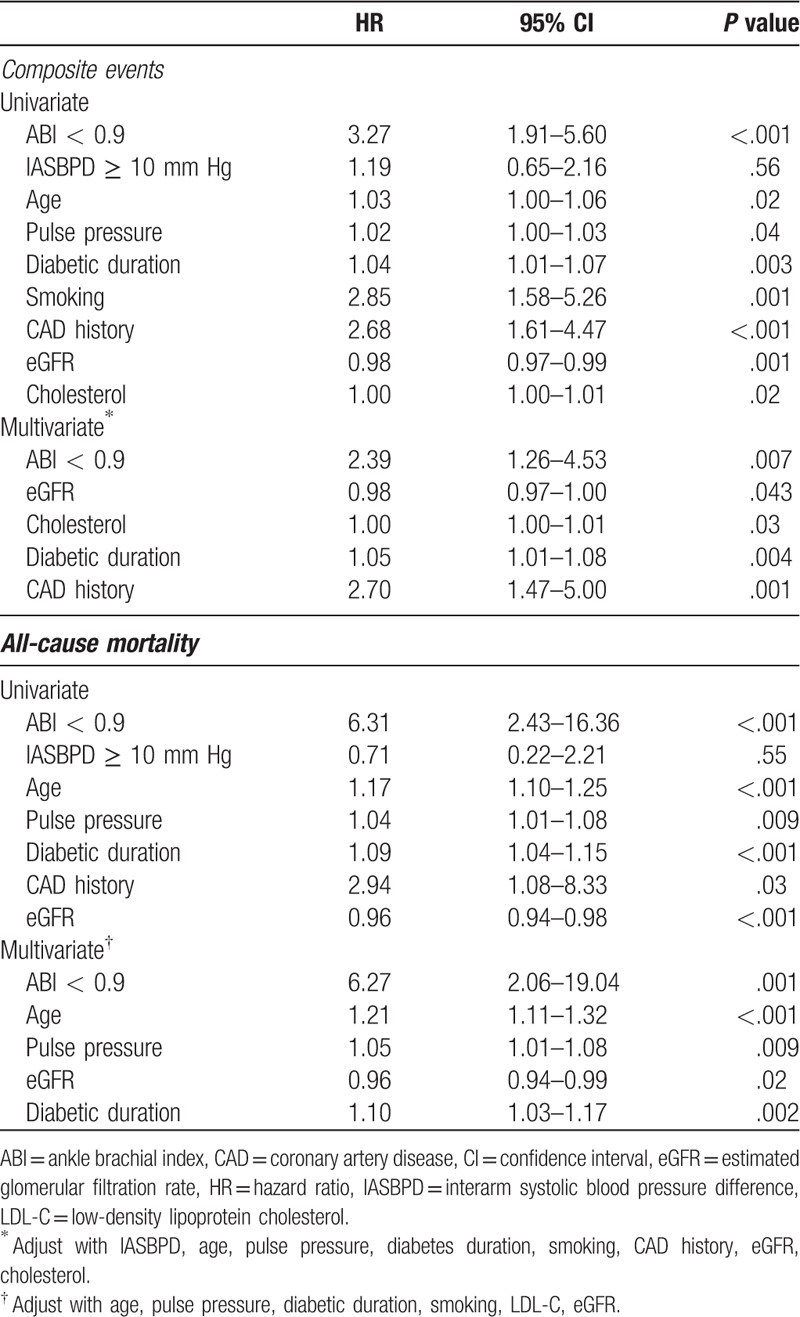
Result of univariate and multivariate Cox proportional hazard model for association of parameters with composite events and all-cause mortality.

### 3.4 Regression analyses of ABI and IASBPD divided into quartiles

We divided all patients into quartiles based on ABI and IASBPD to analyze the risk of clinical outcomes. Table 3 shows the Cox proportional hazard analyses for the associations of ABI and IASBPD in quartiles with composite events and all-cause mortality. Only patients in the lowest quartile of ABI had a significant risk of composite events (HR, 3.27; 95% CI, 1.64–6.49, *P* < .01) and all-cause mortality (HR, 13.26; 95% CI, 1.72–102.71, *P* = .01). However, this association was not found when patients were divided based on IASBPD.

**Table 3:**
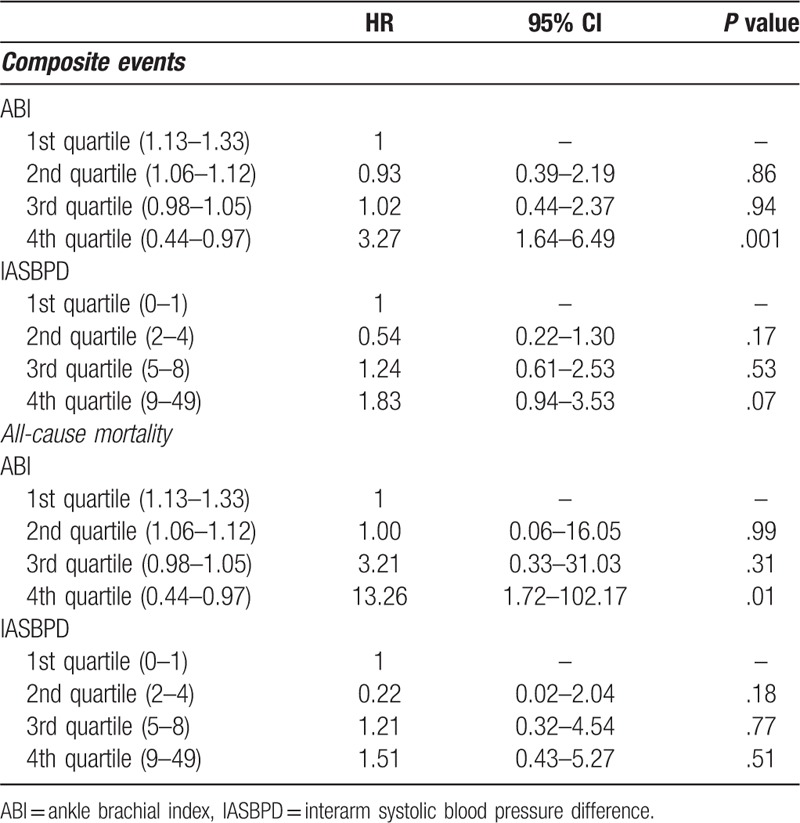
Result of cox proportional hazard model for the associations of quartile of ABI and IASBPD with composite events and all-cause mortality.

## 4 Discussion

In the present study, ABI < 0.9 rather than IASBPD > 10 mm Hg exhibited a significant association with composite events and all-cause mortality in patients with T2DM. The tremendous increment of risk in cardiovascular outcomes was found in the lowest quartile group of ABI. The risk of cardiovascular events increased along with increment in IASBPD; however, the HR of the highest quartile group with IASBPD majorly >10 mm Hg did not reach statistical significance. Neither ABI < 0.9 plus IASBPD ≥ 10 mm Hg (data not shown) nor IASBPD associated with all-cause mortality demonstrated that the non-HDL-c was less important factor to be influenced on the risk of composite events and all-cause mortality in this analysis comparing ABI.

The ABI was found to be the dominant determining factor associated with cardiovascular outcomes in patients with T2DM in the present study ant this finding was consistent with previous studies in patients with diabetes.^[17–19]^ Patients with PAD might receive less-intensive medical therapy according to the Global Registry of Acute Coronary Events study, and a history of PAD was associated with worse cardiovascular outcomes.^[20]^ However, Hanssen et al reported a paradoxical association, which showed no significant difference in the association of ABI with all-cause mortality and cardiovascular mortality between patients with and without diabetes mellitus.^[15]^ The stiffness and calcification of the arteries in patients with diabetes may explain the paradoxical association, and the cut-off ABI value for the diagnosis of peripheral vascular disease might be modified. The theory was evidenced by an epidemiologic study conducted by Yan et al. Even borderline ABI, defined as ABI between 0.90 and 0.99, was associated with retinopathy, macroalbuminuria, chronic kidney disease, and stroke in patients with T2DM.^[21]^ Moreover, ABI also predicts rapid renal function decline, except for major cardiovascular events, in patients with atrial fibrillation.^[22]^ Taken together, though the optimal threshold of ABI for the diagnosis of peripheral vascular disease and prediction of outcomes of diabetes has not been completely clarified, ABI per se is still a predictor of cardiovascular outcomes in patients with T2DM.

Furthermore, ABI plays a complementary role in risk stratification of cardiovascular disease. The Framingham risk score is a well-known validated tool to assess cardiovascular risk factors and identify high-risk individuals among individuals without diabetes.^[23]^ Additional parameters are needed together with the Framingham risk score to assess cardiovascular risk, particularly for asymptomatic individuals with diabetes. ABI was considered as a parameter to improve the accuracy of cardiovascular risk prediction beyond the Framingham risk score.^[24]^ A device can obtain IASBPD and ABI simultaneously, preventing the bias of measurement resulting from discrepancy of different devices or timing of data collection; therefore, direct comparison between ABI and IASBPD was reasonable in the present study. Increased differences in blood pressure between limbs are recognized as the consequence of PAD.^[25,26]^ IASBPD was indicated to be associated with pulse-wave velocity^[27,28]^ and carotid intima medial thickness.^[29]^ However, the IASBPD was not associated with primary or secondary outcomes in our cohort of patients with T2DM. The IASBPD was associated with some parameters, which were associated with cardiovascular outcomes, but the association of IASBPD with cardiovascular outcomes cannot be found because the actual mechanisms of differences of blood pressure between limbs are not completely understood in patients with diabetes. Furthermore, the normal range threshold seems to be changed in different populations, except for elusive mechanisms of IASBPD. Several reports described IASBPD > 10 mm Hg to be associated with elevated cardiovascular event rates, but 15 mm Hg seems to be a more appropriate threshold for diabetes.^[30–32]^ We did not find an association between IASBPD and cardiovascular outcomes in the present study, and even the highest quartile group of IASBPD included patients with IASBPD > 9 mm Hg. In addition, IASBPD was also not associated with all-cause mortality in the previous cohort study.^[33]^ Another possible explanation for neutral effects on outcomes of IASBPD was the influence of medications. Our patients were being treated with several medications including angiotensin converting enzyme inhibitor, angiotensin II receptor blockade, statin, beta-blocker, or antiplatelet drugs. The use of these medications might influence the association between IASBPD and all-cause mortality, but there was no imbalance in the use of specific medications in our study. Further studies to assess the impact of medication on cardiovascular outcomes in diabetes with or without PAD should be conducted in the future.

This study has some limitations. First, this is a retrospective study conducted in a single center in Taiwan; therefore, the selection bias is inevitable, and whether the results can be applied to other ethnicities should be verified by further research. Second, the numbers of patients with diabetes with ABI < 0.9 were relatively too small compared with the general population; however, the prevalence rate is consistent with the present study.^[34]^ Furthermore, multicenter prospective studies are warranted to confirm the conclusion of our study. Finally, the medical histories and cardiovascular events based on medical records might have led to bias in this analysis.

In conclusion, ABI shows a better association with cardiovascular outcomes, including composite cardiovascular events and all-cause mortality, than IASBPD in patients with diabetes. The study findings indicate that routine screening using ABI via noninvasive modality would be recommended in asymptomatic patients with T2DM.

## Acknowledgment

The authors thank the Medical Sciences and Technology Building of Taipei Veterans General Hospital for providing them with an experimental space and facilities.

## Author contributions

Analysis data: LYL, CHC, LHC.

Cases contributor: CMH, JGSW, LYL.

Study design and data collection: LYL, CMH, LHC.

Writing the manuscript: LYL, CHC, LHC.

**Data curation:** Justin G.S. Won.

**Methodology:** Chia-Huei Chu.

**Supervision:** Chii-Min Hwu, Harn-Shen Chen.

**Writing – original draft:** Liang-Yu Lin.

**Writing – review & editing:** Li-Hsin Chang.

Li-Hsin Chang orcid: 0000-0002-6969-5432.
